# The Subantarctic Rayadito (*Aphrastura subantarctica*), a new bird species on the southernmost islands of the Americas

**DOI:** 10.1038/s41598-022-17985-4

**Published:** 2022-08-26

**Authors:** Ricardo Rozzi, Claudio S. Quilodrán, Esteban Botero-Delgadillo, Constanza Napolitano, Juan C. Torres-Mura, Omar Barroso, Ramiro D. Crego, Camila Bravo, Silvina Ippi, Verónica Quirici, Roy Mackenzie, Cristián G. Suazo, Juan Rivero-de-Aguilar, Bernard Goffinet, Bart Kempenaers, Elie Poulin, Rodrigo A. Vásquez

**Affiliations:** 1 Cape Horn International Center (CHIC), Parque Etnobotánico Omora, Universidad de Magallanes, Puerto Williams, Chile; 2 Sub-Antarctic Biocultural Conservation Program, Department of Philosophy and Religion and Department of Biological Sciences, University of North Texas, Denton, TX USA; 3 Department of Biology, University of Fribourg, Fribourg, Switzerland; 4 Department of Behavioural Ecology and Evolutionary Genetics, Max Plank Institute for Ornithology, Seewiesen, Germany; 5 Departamento de Ciencias Ecologicas, Facultad de Ciencias, Universidad de Chile, Santiago, Chile; 6 Departamento de Ciencias Biológicas y Biodiversidad, Universidad de Los Lagos, Osorno, Chile; 7 Instituto de Ecología y Biodiversidad, Santiago, Chile; 8 AvesChile (Unión de Ornitólogos de Chile), Santiago, Chile; 9 Smithsonian National Zoo and Conservation Biology Institute, Conservation Ecology Center, 1500 Remount Rd, Front Royal, VA 22630 USA; 10 Departamento de Zoología, CRUB Universidad Nacional del Comahue-CONICET, Bariloche, Argentina; 11 Centro de Investigación Para la Sustentabilidad, Facultad de Ciencias de la Vida, Universidad Andres Bello, Santiago, Chile; 12 Department of Animal Ecology and Systematics, Justus Liebig University Giessen, Giessen, Germany; 13 Department of Ecology and Evolutionary Biology, University of Connecticut, Storrs, CT 06269 USA; 14 Millennium Institute Biodiversity of Antarctic and Subantarctic Ecosystems (BASE), Facultad de Ciencias, Universidad de Chile, Santiago, Chile

**Keywords:** Biodiversity, Conservation biology, Ecological genetics, Evolutionary ecology

## Abstract

We describe a new taxon of terrestrial bird of the genus *Aphrastura* (rayaditos) inhabiting the Diego Ramírez Archipelago, the southernmost point of the American continent. This archipelago is geographically isolated and lacks terrestrial mammalian predators as well as woody plants, providing a contrasted habitat to the forests inhabited by the other two *Aphrastura *spp. Individuals of Diego Ramírez differ morphologically from *Aphrastura spinicauda*, the taxonomic group they were originally attributed to, by their larger beaks, longer tarsi, shorter tails, and larger body mass. These birds move at shorter distances from ground level, and instead of nesting in cavities in trees, they breed in cavities in the ground, reflecting different life-histories. Both taxa are genetically differentiated based on mitochondrial and autosomal markers, with no evidence of current gene flow. Although further research is required to define how far divergence has proceeded along the speciation continuum, we propose *A. subantarctica* as a new taxonomic unit, given its unique morphological, genetic, and behavioral attributes in a non-forested habitat. The discovery of this endemic passerine highlights the need to monitor and conserve this still-pristine archipelago devoid of exotic species, which is now protected by the recently created Diego Ramírez Islands-Drake Passage Marine Park.

## Introduction

**Figure 1:**
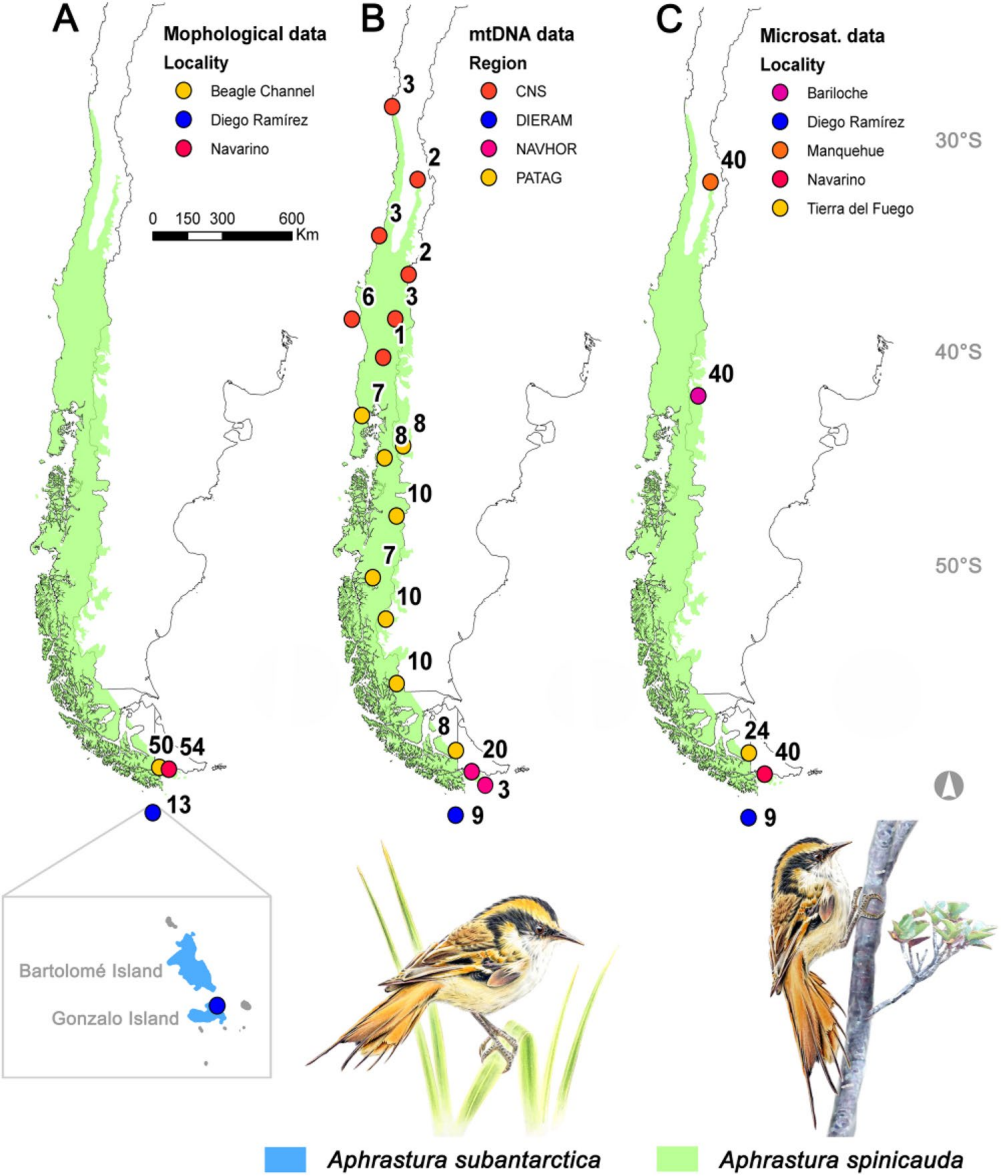
Study areas for the morphological and genetic characterization of *Aphrastura spinicauda* and *A. subantarctica*. The distribution range of the nominal species is shown in light green, and the new taxonomic group from the Diego Ramírez Archipelago in light blue. (**A**) Sampling sites for morphology. (**B**) Sampling sites for mtDNA. (**C**) Sampling sites for microsatellite markers. The numbers correspond to the sample size. The names of the colored sites follow the methods description, and Tables 1 and 2. Bird illustrations by Mauricio Alvarez Abel.

The genus *Aphrastura* (Passeriformes: Furnariidae) is endemic to southwestern South America and includes two allopatric species: the Thorn-tailed Rayadito (*A. spinicauda*) that inhabits the temperate forest biome of South America^1^, and the Masafuera Rayadito (*A. masafuerae*) whose distribution is limited to the misty tree fern forests of the oceanic Alejandro Selkirk Island^2,3^. This island has an area of only 85 km^2^ and is part of the Juan Fernández Archipelago, separated from the continent by 670 km. Unlike *A. masafuerae*, *A. spinicauda* has a broad distribution along the entire latitudinal range of the South American temperate forests’ biome^4^. It inhabits deciduous and evergreen forest types ranging from north-central Chile to the extreme south of Chile and Argentina^5–7^. Its northernmost population is found in the evergreen relict forest of Fray Jorge National Park (30° 30′ S), whilst its southerly population reaches the world’s southernmost forests in the Cape Horn Biosphere Reserve (CHBR) (56° S)^7^. However, a putative population of *A*. *spinicauda* also occurs on the Diego Ramírez Archipelago (hereafter Diego Ramírez) (56° 32′ S)^8–10^ (Fig. 1). Whether this population differs morphologically, ecologically, and genetically from its continental relatives remains unexplored.

**Figure 2:**
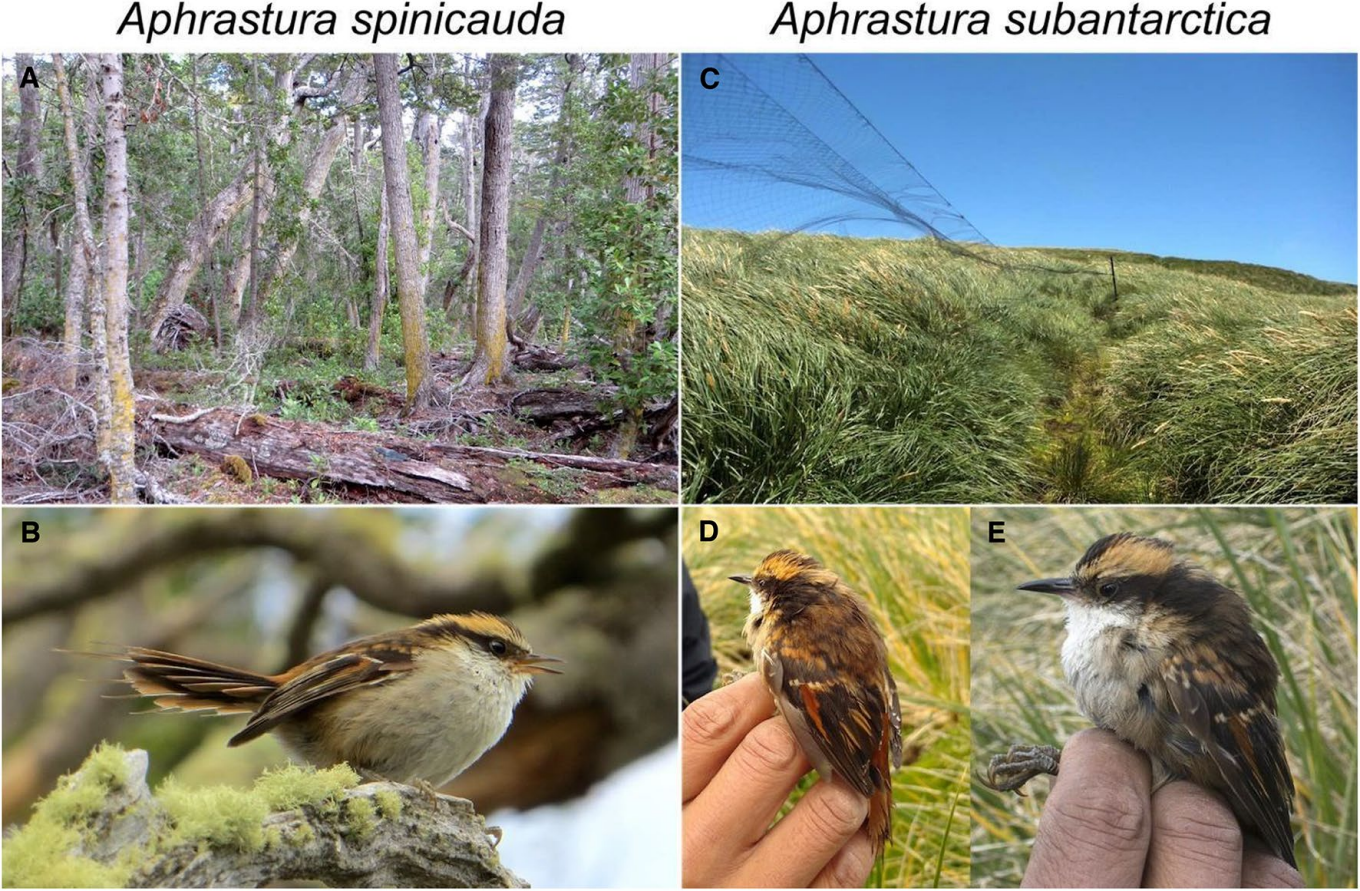
Habitat characteristics and individual appearance of two populations of *Aphrastura*. (**A**) Forest habitat on Navarino Island. (**B**) An individual of the thorn-tailed rayadito (*Aphrastura spinicauda*) from Navarino Island. (**C**) Tussock (*Poa flabellata*) habitat on Gonzalo Island, Diego Ramírez Archipelago, with a mist net. (**D**,**E**) Individuals of the proposed new species *A. subantarctica* from Gonzalo Island. Images by Omar Barroso.

The Diego Ramírez is the southernmost point of the South American continent. While emerging only ca. 100 km southwest of Cape Horn on the margin of the continental shelf, it is separated from it by one of the world’s roughest seas in the northern section of the Drake Passage, with harsh climatic conditions and difficult access from the continent^11^. The archipelago of small islets, rocks, and reefs provides a total land area of only 79 hectares^12^. The strong barrier to dispersal and the small size likely severely limit effective population migration to Diego Ramírez, thereby promoting the emergence of a fauna with zoogeographic novelties^8^. Their terrestrial avifauna is mainly limited to breeding seabirds, but despite the complete absence of woody plants (Fig. 2), it also includes passerines^9^, such as a resident population of *Aphrastura spinicauda*^4^. This population is currently considered to be conspecific with populations from the mainland (i.e., hereafter, we refer to them as *Aphrastura* populations from either the continent or Diego Ramírez). The geographic isolation and entirely herbaceous vegetation of Diego Ramírez raises the question whether its *Aphrastura* population constitutes a different taxonomic unit. This possibility is supported by the fact that two other populations of *A*. *spinicauda* that occur on islands have already been recognized as subspecies, based on their morphological distinctiveness, in terms of color and size, namely *A. s. fulva* from Chiloé Island (42° S) and the Chonos Archipelago (44° S), and *A. s. bullocki* from Mocha Island (38° S)^4,13^ (Supplementary Information Appendix 1). However, in contrast to the population inhabiting Diego Ramírez, both subspecies are geographically closer to the continental population and inhabit similar forest habitats.

The two currently recognized species of *Aphrastura* (*A. spinicauda* and *A. masafuerae*) are restricted to forest ecosystems, including forest edges and surrounding woody vegetation^14–17^. The association with such habitats is striking in the northern range of *A. spinicauda*, where it is confined to forest patches and birds rarely venture into the arid shrubland^18,19^. Individuals usually forage for adult insects and larvae in the bark of trunks and branches, but they also glean them from the canopy and understory vegetation^20^. *A. spinicauda* typically occurs in *Nothofagus*-dominated forests, where they are one of the most abundant bird species, nesting in narrow cavities of old-growth trees that provide protection from rain, strong wind, and predators^7^. Given their strong association to forests, it is noticeable to find a population of *Aphrastura* on Diego Ramírez, whose vegetation is completely devoid of trees and other woody plants^21–23^.

Gene flow between the Diego Ramírez and the continental populations of *Aphrastura* is absent^10^, but whether this led to phenotypic divergence remains unknown. Here, we present an integrative taxonomic study combining morphological, ecological, and genetic data to clarify the taxonomic status of the Diego Ramírez population. We propose that this population is sufficiently distinct from continental populations to warrant taxonomic recognition, as the endemic *Aphrastura subantarctica*.

## Methods

### Study area

The Diego Ramírez Archipelago (56° S) is subject to an oceanic climate, characterized by an even rainfall throughout the year, low annual temperature range, and strong western winds^21,22^. The average annual rainfall is 1500 mm, and mean annual temperature is 5.6 °C, with August being the coldest month (3.3 °C on average) and March the warmest month (7.5 °C)^24^. The vegetation lacks trees and shrubs, and is dominated by tall (> 1 m) tussocks (*Poa flabellata*), a herbaceous plant that does not offer cavities for nesting^8,22^ (Fig. 2). Yet, dense aggregations of tussocks provide shelter from the strong and constant winds, such that the wind velocity drops to near zero at ground level. Also, inside the tussocks, temperatures can be up to 5 °C higher than above the plants^23^, creating a protected microhabitat that is used by the *Aphrastura* birds. Gonzalo Island is the second largest island of Diego Ramírez (38 ha), and is also an important nesting site for the Black-browed (*Thalassarche melanophrys*) and Grey-headed albatross (*T. chrysostoma*) (> 11,000 and > 5000 pairs, respectively^25^), which nest in openings of the tussock grassland.

To quantify morphological differences, we surveyed several sites on the north-western arm of the Beagle Channel on Tierra del Fuego Island and on Navarino Island (Fig. 1)^26,27^. These sites represent the southern limit of continental *Aphrastura* populations and are the closest forested habitat to Diego Ramírez. These areas are dominated by evergreen forests of Magellan's beech (*Nothofagus betuloides*) and Winter's bark (*Drimys winteri*)^27,28^. The influence of the ocean moderates thermal fluctuations, allowing the growth of a lush broadleaf evergreen forest. At the eastern end of the Beagle Channel, Navarino Island has an oceanic climate with a reduced annual rainfall of 460 mm, and snowfall mostly from fall to spring^28^. On this island, the high deciduous beech (*N. pumilio*) co-dominates with Magellan's beech, generating mixed forest of evergreen and deciduous trees. *Nothofagus* trees reach heights of 20 m and their straight trunks often have natural cavities that are used by the *Aphrastura* population for nesting^7^. Samples for the morphological analysis included individuals captured on the sites above mentioned (Fig. 1). For the genetic analyses, additional samples were obtained from across the distributional range of *A. spinicauda* (see below).

### Bird capture and measurements

We obtained measurements of *Aphrastura* from Diego Ramírez and two areas of the CHBR, i.e., the northwest arm of the Beagle Channel and Navarino Island (Fig. 1). On Diego Ramírez, we sampled the population on Gonzalo Island (56° 32′ S, 68° 42′ W) on three expeditions: (1) November 29–December 2, 2016; (2) July 20–22, 2017; and (3) March 28—April 1, 2018. In the Beagle Channel, we sampled at four sites: (1) Pia sound (54° 47′ S, 69° 35′ W), (2) German glacier (54° 53′ S, 69° 24′ W), and (3) Olla Cove (54° 56′ S, 69° 09′ W), and (4) Contreras Cove (54° 50′ S, 68° 05′ W). The first three sites were visited between January 20 and 30 in 2015, and Contreras Cove from April 1 to 8, 2017. For Navarino Island, we used data from the Long-Term Ornithological Study Program^7^ at two sites: (5) the Omora Park (54° 06′ S, 67° 39′ W) collected from December 2015 to January 2016, and on April 1, 2018 (to compare with the 2018 Diego Ramírez expedition), and at (6) Guerrico (54° 54′ S, 67° 51′ W) in March 2015 and 2016. To avoid seasonal morphological variability, we restricted the morphological analysis to data obtained during the austral summer (November to April).

In all sites, birds were captured using 2–6 mist nets of mesh size 30 mm and 2.4 × 12 m long each. We operated the nets for 6-h periods after sunrise. Given the difficulties to operate the nets on Diego Ramírez (strong winds and dense herbaceous vegetation), we cleared a transect in the tussock to set the mist nets (Fig. 2). The nets were checked every 15–20 min. Birds were aged, banded, measured, and released following a protocol designed for the Ornithological Studies Program of the Omora Park^7^. We measured wing length, tarsus length, tail length, beak length and beak width using a caliper (0.01 mm precision), and we determined body mass using a digital scale (0.1 g precision). The same person (OB) performed all the measurements. Blood samples were obtained by puncturing the brachial vein and stored on FTA^®^ cards for subsequent genetic analyses.

### Observations of habitat use on Diego Ramírez

To compare the well-known habitat used by other *Aphrastura* populations, we recorded the habitat use of the Diego Ramírez *Aphrastura* individuals. In each austral spring and summer between 2010 and 2021, one observer (CGS) recorded all individuals along two transects (1.02 km length each) starting from the shoreline to the highest ridge of Gonzalo Island (129 m above sea level, masl). These transects included locations with different tussock density (50–100% coverage) and plant heights (0.4–2.0 m), as well as other bare sites with rocky or muddy substratum. These habitats are used as nesting sites by black-browed and grey-headed albatrosses. The transects also covered areas used by other surface-nesting seabirds, like the rockhopper (*Eudyptes chrysocome*), and macaroni penguin (*E. chrysolophus*), as well as burrowing seabird species, such as the Magellanic penguin (*Spheniscus magellanicus*), blue petrel (*Halobaena caerulea*) and the common diving petrel (*Pelecanoides urinatrix*). Nesting colonies of these sea birds modify the habitat in a way that may be relevant for the Diego Ramírez *Aphrastura* population. Based on the transects, we calculated the relative number of *Aphrastura* (birds/km) on each island. Based on all observations, we qualitatively described the habitat type used by *Aphrastura* (i.e., tussock coverage (%) and height (m), their nesting sites, and their behavior (foraging, territory defense, and their association with other bird species)). In addition, one observer (OB) carried out opportunistic observations during mist-netting, observing birds when walking in different directions to cover an area of approximately 0.16 km^2^ (~ 42% of the surface of Gonzalo Island).

### Data analysis

#### Morphological analysis

To avoid age-related morphological differences, we only used measurements from adult individuals for this analysis. We compared trait values across the sites using a Kruskal–Wallis rank sum test and Conover's non-parametric all-pairs comparison test for a posteriori analysis (α = 0.05). To examine overall morphological differences among populations, we performed a Principal Components Analysis (PCA) including z scores for all measured variables. All analyses were performed using R^29^.

#### Molecular analysis and sequencing

We used blood samples from populations that covered the entire distributional range of *A. spinicauda*, from Fray Jorge National Park in north-central Chile to Navarino and Horn islands, and including nine individuals from Diego Ramírez (Fig. 1; Table 1). Total DNA was isolated from 100 µg of blood using the DNeasy Blood and Tissue kit from Qiagen (Germany) following the manufacturer’s protocol. PCR amplifications and bidirectional sequencing (Macrogen Inc., South Korea) of the mitochondrial cytochrome b gene (cytb) were carried out followed Gonzalez and Wink^30^. Consensus sequences of forward and reverse sequences were aligned using Pro-Seq 2.91^31^ against reference sequences^30^, and were visually checked.

**Table 1:** Measures of genetic diversity based on mtDNA in different *Aphrastura* geographic groups.

Geographic group	n	K	Kr	S	H	π	D	Fu
CNS	20	2	1.45	1	0.10	0.10	− 1.16	− 0.88
PATAG	68	11	2.92	11	0.43	0.57	− **2.08**	− **10.02**
NAVHOR	23	4	2.67	3	0.39	0.42	− 1.24	− **1.84**
DIERAM	9	1	1	0	0.00	0.00	0	0
Total	120	14	−	14	0.45	0.56	− **2.10**	− **3.30**

*CNS* Center, north and south, *PATAG* Patagonia (Chiloé to Tierra del Fuego), *NAVHOR* Navarino and Horn islands, *DIERAM* Diego Ramírez, *N* sample size, *K* number of haplotypes, *Kr* number of haplotypes based on rarefaction curves to compare diversity in geographic groups with different sample sizes (see “Methods” for details), *S *number of polymorphic sites, *H* heterozygosity, *π* average number of nucleotide differences between pairs of sequences,* D* Fu and Li’s D neutrality test, *Fu* Fu’s neutrality test. Statistically significant values are indicated in bold.

#### Phylogeographic analysis

We used Arlequin 3.5.1.2^32^ to estimate the number of haplotypes and polymorphic sites, gene diversity, differences between pairs of sequences (π) and nucleotide diversity (π per nucleotide site). For comparison of the four geographic groups (Fig. 1; Table 1), we used a rarefaction analysis with PAST^33^ to adjust for unequal sample sizes. We generated a haplotype network using the median-joining approach method^34^ implemented in Network 4.6. Using Arlequin 3.5.1.2^32^, we estimated indices of genetic differentiation among geographic units based on both allele frequencies and pairwise nucleotide differences (pairwise *F*_ST_ and Ф_ST_, respectively). We tested for phylogeographic structure by comparing G_ST_ and N_ST_ coefficients using permutation tests implemented in PERMUT^35^. To infer the spatial genetic structure of *Aphrastura* populations, we used an analysis of molecular variance (AMOVA)^36^, defining groups of populations that are geographically homogeneous and maximally differentiated from each other.

#### Population genetic analysis

We performed further genetic analyses based on nuclear microsatellite markers to check the conclusions from the mitochondrial phylogeographic approach. We analyzed an additional sub-sample of 153 individuals from Central Chile, Southern Chile and Argentina, and Diego Ramírez (Fig. 1; Table 2). These individuals were originally genotyped for another study led by EB-D^10^, using 12 autosomal polymorphic loci^37^. The marker panel consisted of seven species-specific markers and five cross-species amplifying markers (see^10,38^). None of the loci showed evidence for deviations from Hardy–Weinberg equilibrium (all p > 0.1) and all had null allele frequencies < 0.05. We calculated measures of genetic diversity per population using the *hierfstat* package^39^ in R. To summarize overall genetic variability among individuals and to preliminarily assess genetic structure, we used a PCA as implemented in the R package *adegenet*^40^. To estimate the optimal (most likely) number of genetic clusters that were present in our sample, we used the ‘snapclust’ method in *adegenet*. This method relies on maximizing the likelihood of a number of panmictic populations by combining model-based and geometric approaches^41^. The best-supported number of groups was determined using the Akaike Information Criterion (AIC).

**Table 2:** Localities, sample sizes and measures of genetic diversity based on 12 microsatellite loci in five *Aphrastura* populations.

Locality	Latitude/longitude	n	Na	Na (rarefied)*	Np	Ho	He	F_IS_
Manquehue	33.35° S/70.57° W	40	8.42 (1.01)	6.23 (0.64)	0.42 (0.15)	0.72 (0.06)	0.73 (0.06)	0.009
Bariloche	41.15° S/71.38° W	40	12.08 (1.76)	7.19 (0.84)	2.08 (0.72)	0.75 (0.06)	0.76 (0.05)	0.013
Tierra del Fuego	54.11° S/69.36° W	24	9.66 (1.40)	6.51 (0.78)	0.58 (0.26)	0.74 (0.06)	0.74 (0.05)	− 0.008
Navarino Island	54.94° S/67.64° W	40	9.66 (1.26)	6.12 (0.65)	0.58 (0.26)	0.75 (0.04)	0.74 (0.04)	− 0.028
Diego Ramírez	56.52° S/68.71° W	9	2.25 (0.45)	2.25 (0.45)	0.08 (0.08)	0.22 (0.08)	0.25 (0.09)	0.026

*N* sample size, *Na* number of alleles, *Np* number of private alleles, *Ho* observed heterozygosity, *He* expected heterozygosity, *F*_*IS*_ within-population inbreeding coefficient. Mean values and standard errors over 12 microsatellite loci are reported.

*The allelic richness for the minimum number of individuals genotyped in any population (times 2).

The resulting genetic clusters were subsequently used for population structure analyses. First, we used GenAlex 6.5^42^ to calculate pairwise G-statistics for the genetic clusters identified, implementing both the Nei’s standardized index (*G’*_*ST(Nei)*_) and the Hedrick’s standardized index corrected for small samples (*G″*_*ST*_). Second, we calculated the posterior membership probabilities of all sampled individuals for the newly identified genetic clusters, using the probability of assignment obtained from the ‘snapclust’ method (see^41^). Finally, we performed a complementary analysis for inferring individual membership to each genetic group using GeneClass2^43^ to identify first-generation migrants. Detection of migrants was carried out using Paetkau et al.’s^44^ criterion for likelihood computation (*L*_*h*_) and Paetkau et al.’s^45^ resampling method for probability calculation, setting the default frequency for missing alleles at 0.01 and using 10 replicates with 100 simulated individuals each (α = 0.01).

### Permits and ethical statement

The sampling methodology was approved by the Ethics Committee of the Sciences Faculty of the University of Chile, following guidelines from the Biosecurity Manual of CONICYT (version 2008) and Chilean law N°20380 about animal protection. Full permissions for sampling and animal ethics approval were granted by the Servicio Agrícola y Ganadero (SAG; permits N° 5158/2016, 8195/2016, 4209/2017, and 2667/2018), the Corporación Nacional Forestal (CONAF; Resolution N° 711/2014), Chile; and the Administración de Parques Nacionales (APN-DRPN research permit N° 1405), Argentina. This manuscript complies with the Animal Research: Reporting of In Vivo Experiments (ARRIVE) guidelines^46^.

## Results

### Comparison of morphology

**Figure 3:**
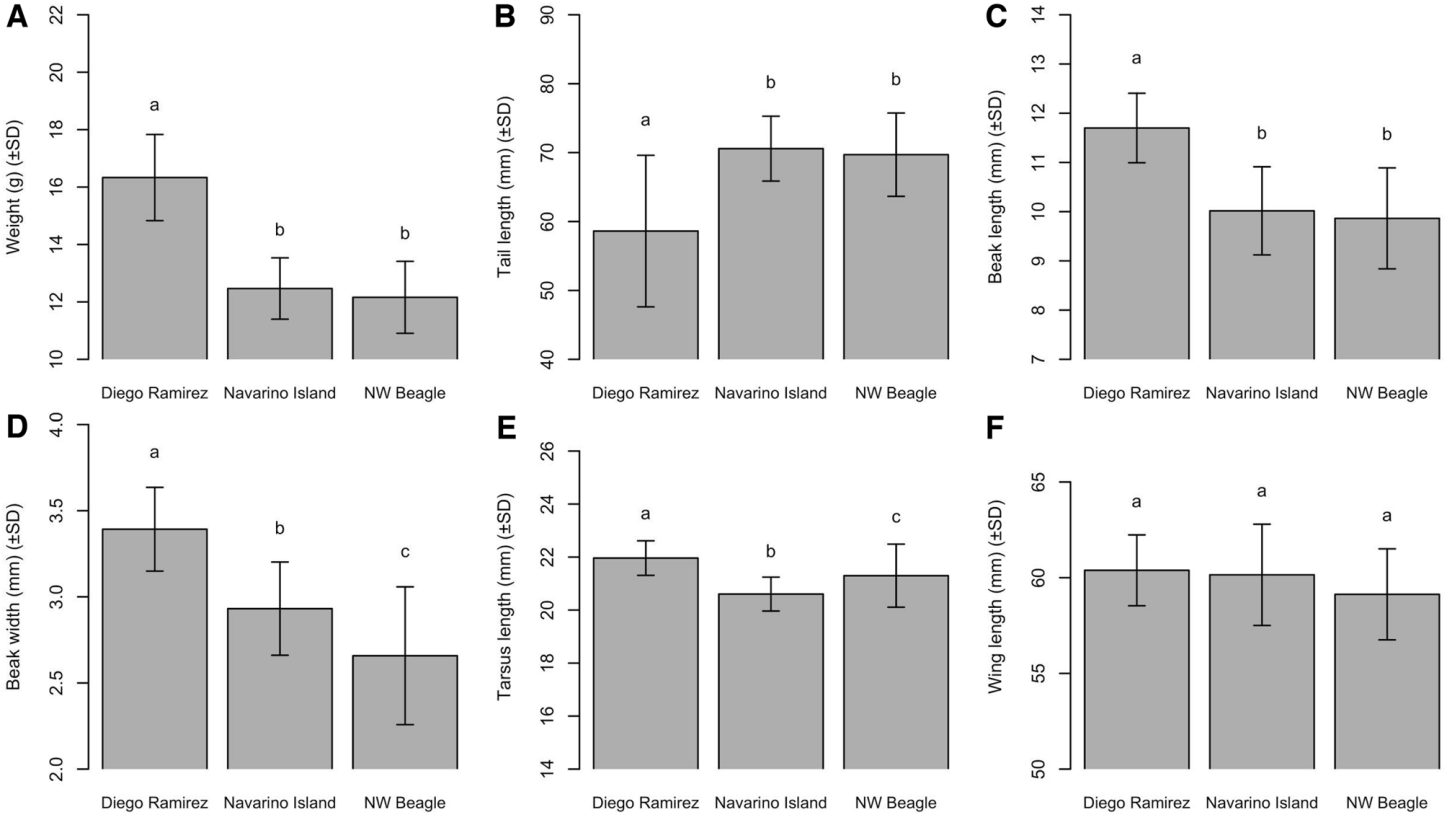
Comparison of body weight (**A**), tail length (**B**), beak length (**C**), beak width (**D**), tarsus length (**E**), and wing length (**F**) among *Aphrastura* populations. Metrics (means ± SD) of birds from the northwest arm of the Beagle Channel (NW Beagle, n = 50), Navarino Island (n = 54), and the population of the proposed new species *A. subantarctica* on Gonzalo Island, Diego Ramírez (n = 13). Lowercase letters indicate statistically significant differences at an alpha of 0.05.

**Figure 4:**
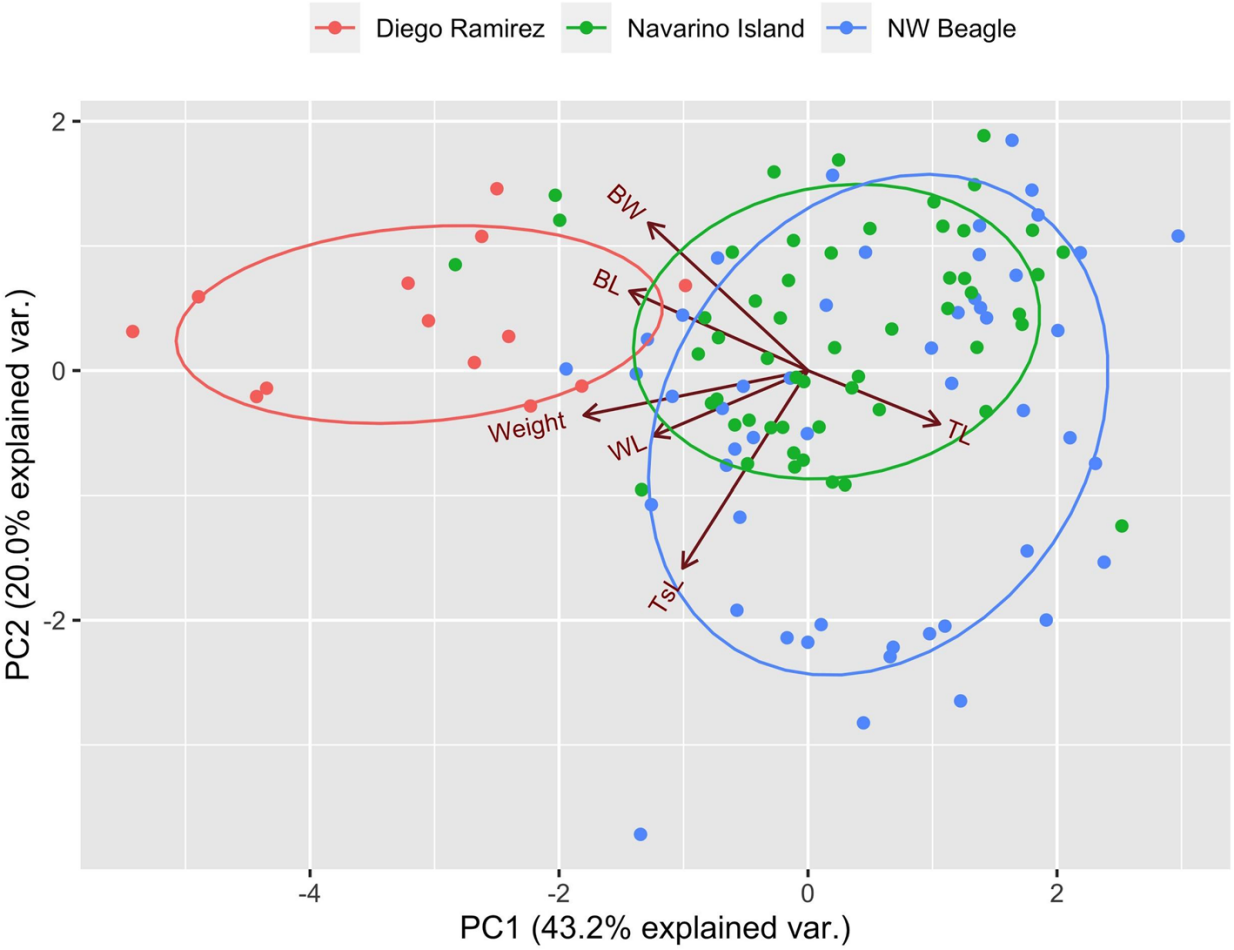
Principal component analysis of morphological variation of populations of thorn-tailed Rayadito (*Aphrastura spinicauda*) in the northwest arm of the Beagle Channel (blue) and Navarino Island (green), and for the population of the proposed new species *A. subantarctica* on Gonzalo Island, Diego Ramírez (red). Plotted are the first two components from a principal component analysis based on 117 measured individuals. *WL* wing length, *TsL* tarsus length, *TL* tail length, *BL* beak length, *BW* beak width, *Weight* body mass.

We captured and measured 117 adult individuals of *Aphrastura* in the NW Beagle Channel (n = 50), Navarino Island (n = 54), and Diego Ramírez (n = 13). Morphological differences among populations were statistically supported for weight, tail length, tarsus length, beak length, and beak width (all p < 0.05, Fig. 3A–E), but not for wing length (p > 0.05, Fig. 3F). Birds from the Diego Ramírez population were significantly heavier and larger (with a longer and wider bill and longer tarsi), but they had a significantly shorter tail than birds from the other two populations (Fig. 3A–E). The first two axes of the PCA explained 63.2% of the variance in morphological traits (PC1 = 43.2%, PC2 = 20.0%). The PCA analysis shows that the *Aphrastura* populations of the Beagle Channel and Navarino Island overlap in body dimensions, whereas the individuals of the Diego Ramírez population form a clearly separate cluster (Fig. 4).

### Behavior and habitat use on Diego Ramírez

We estimated the relative abundance on Gonzalo Island as 47.1 birds/km (~ 48 birds per transect). Birds of Diego Ramírez typically performed short and low flights, rarely moving above the cover of the tussock grass. On Gonzalo Island, individuals always remained in the wind-protected habitat provided by the tall grass (< 2 m), except for brief horizontal movements to and from more open foraging areas.

We observed 10 nesting sites of *Aphrastura* on Gonzalo Island. Two of them were found in the areas with a higher concentration of breeding seabirds, which were close to the coast (11 masl), characterized by 50% coverage of small tussocks (~ 0.4 m high) and a rocky substrate. Three nesting sites were found in the higher and wind-exposed areas of the island (> 100 masl), but sheltered by 100% tussock coverage. Yet, most nesting sites (n = 5) were observed in the lower and medium altitude zones, particularly in the presence of protected creeks and areas with tussocks at least 1 m high. These five nesting sites were composed of burrows with single entrances. Four of these burrows were generated and abandoned by underground nesting seabirds, and one was a natural soil cavity. One *Aphrastura* nest was located below an active nest of an incubating Grey-headed albatross (for details see Supplementary Information Appendix 2).

On Gonzalo Island, *Aphrastura* typically foraged about 10–30 m away from the nesting site, capturing invertebrates. Foraging areas were covered by dense tussock or were located in the periphery of the tussock-matrix. We recorded pairs or groups of up to four individuals foraging together. Birds responded to a human intruder (the observer) by active flapping displays, vertical movements between the ground and the tussock canopy, and mobbing calls that started from 25 to 15 m away from the observer and involved pairs or groups of up to five individuals.

### Phylogeographic patterns

We obtained 471 bp *cytb* sequences from 120 individuals across the distributional range of *A. spinicauda* (see sample sizes in Fig. 1)*.* Overall mitochondrial genetic diversity was low, revealing a short genealogy of the *cytb* gene. Genetic diversity was larger in populations from southern Chile between 42 and 53° S (from Chiloé to Punta Arenas), compared to that of populations in the center and the north of the distribution, and also of the populations in the extreme southern part of the distribution (Navarino and Horn islands; Table 1). Noticeably, based on pairwise *F*_ST_ and Ф_ST_ values, the Diego Ramírez population is strongly and significantly separated from all other populations, which exhibit little structure across the distribution range (from Fray Jorge in the north to Horn Island in the south; Table 3). The AMOVA showed that 69% of the total genetic variance was explained by clustering Diego Ramírez apart from all the other geographic groups (Table 4). We also found a strong phylogeographic structure between Diego Ramírez and the other groups, supported by N_ST_ coefficients (0.537 ± 0.136) being higher than G_ST_ values (0.320 ± 0.141; p < 0.001).

**Table 3:** Measures of genetic distance based on mtDNA between *Aphrastura* geographic groups.

Geographic groups	CNS	PATAG	NAVHOR	DIERAM
CNS	–	− *0.00017*	*0.025*	***0.93***
PATAG	− 0.0013	–	− *0.0025*	***0.66***
NAVHOR	0.020	− 0.0051	–	***0.73***
DIERAM	**0.93**	**0.63**	**0.69**	–

*CNS* Center, north and south, *PATAG* Patagonia (Chiloé to Tierra del Fuego), *NAVHOR* Navarino and Horn islands, *DIERAM* Diego Ramírez. Shown are pairwise *F*_ST_ values (above the diagonal, in italics) and Ф_ST_ values (below the diagonal). Statistically significant values are shown in bold (all p < 0.005).

**Table 4:** Analysis of Molecular Variance (AMOVA) based on mtDNA comparing the *Aphrastura* population from Diego Ramírez to populations from other regions (see Table 1).

Source of variation	d.f	Sum of squares	Variance components	Percentage of variation explained
Among groups	1	8.087	0.473	**68.9**
Among populations within groups	2	0.465	0.00065	0.09
Within populations	116	24.706	0.213	31.0
Total	119	33.258	0.686	

Fixation Indices: FSC = 0.0030, FST = 0.69, FCT = 0.69. Statistically significant values are highlighted in bold.

**Figure 5:**
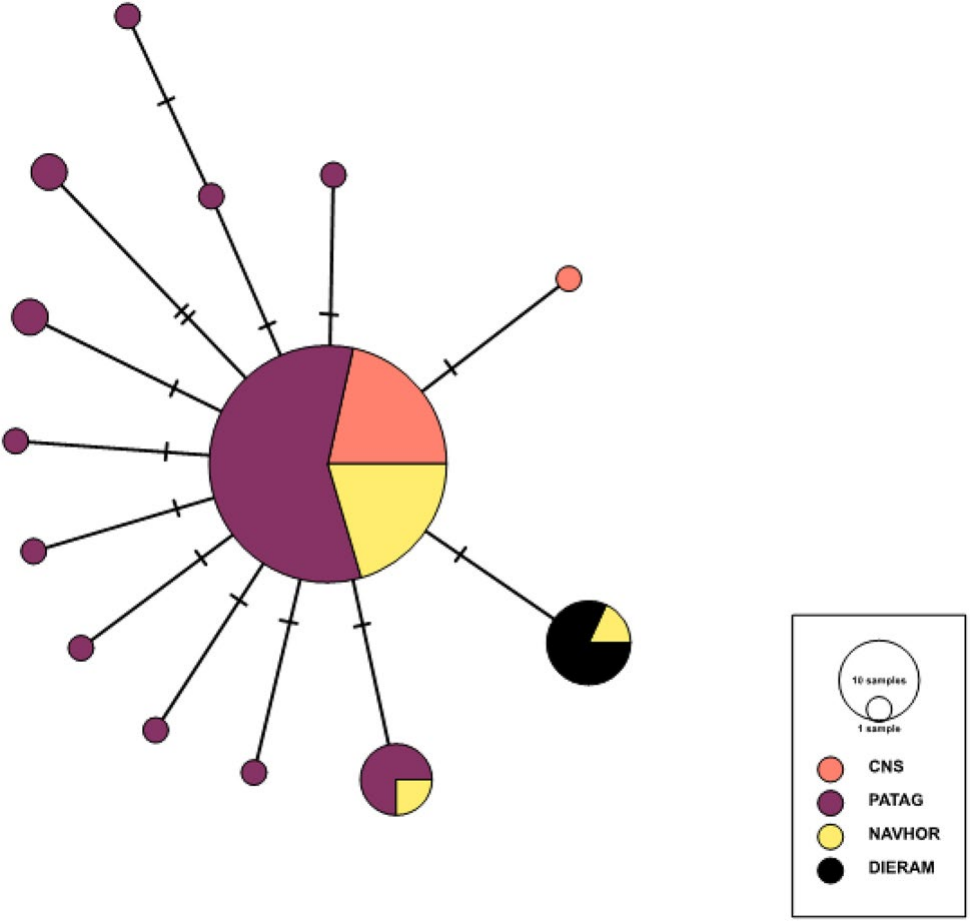
Mitochondrial haplotype network of *Aphrastura* geographic groups. Each circle in the network corresponds to a different haplotype, the size of the circles corresponds to haplotype frequencies, and the colors correspond to the different geographical groups. *CNS* Central, north and south, *PATAG* Patagonia (Chiloé to Tierra del Fuego), *NAVHOR* Navarino and Horn islands, *DIERAM* Diego Ramírez (representing the proposed new species *A. subantarctica*).

Individuals sampled from Diego Ramírez shared the exact same haplotype, which differed by one mutation from the most dominant haplotype found in *A. spinicauda*. The Diego Ramírez haplotype is also present on Horn Island (1 out of 3 individuals) and in low frequency in Navarino Island (1 out of 20 individuals) (Fig. 5).

### Population genetic structure

Based on the genotypes of 153 individuals at 12 polymorphic microsatellite loci (see sample sizes in Fig. 1), genetic diversity was relatively high in all sampled populations, except the one from Diego Ramírez (Table 2). The latter exhibited the lowest heterozygosity, the highest within-population inbreeding coefficient, and the lowest allelic richness, both before and after rarefaction (Table 2).

**Figure 6:**
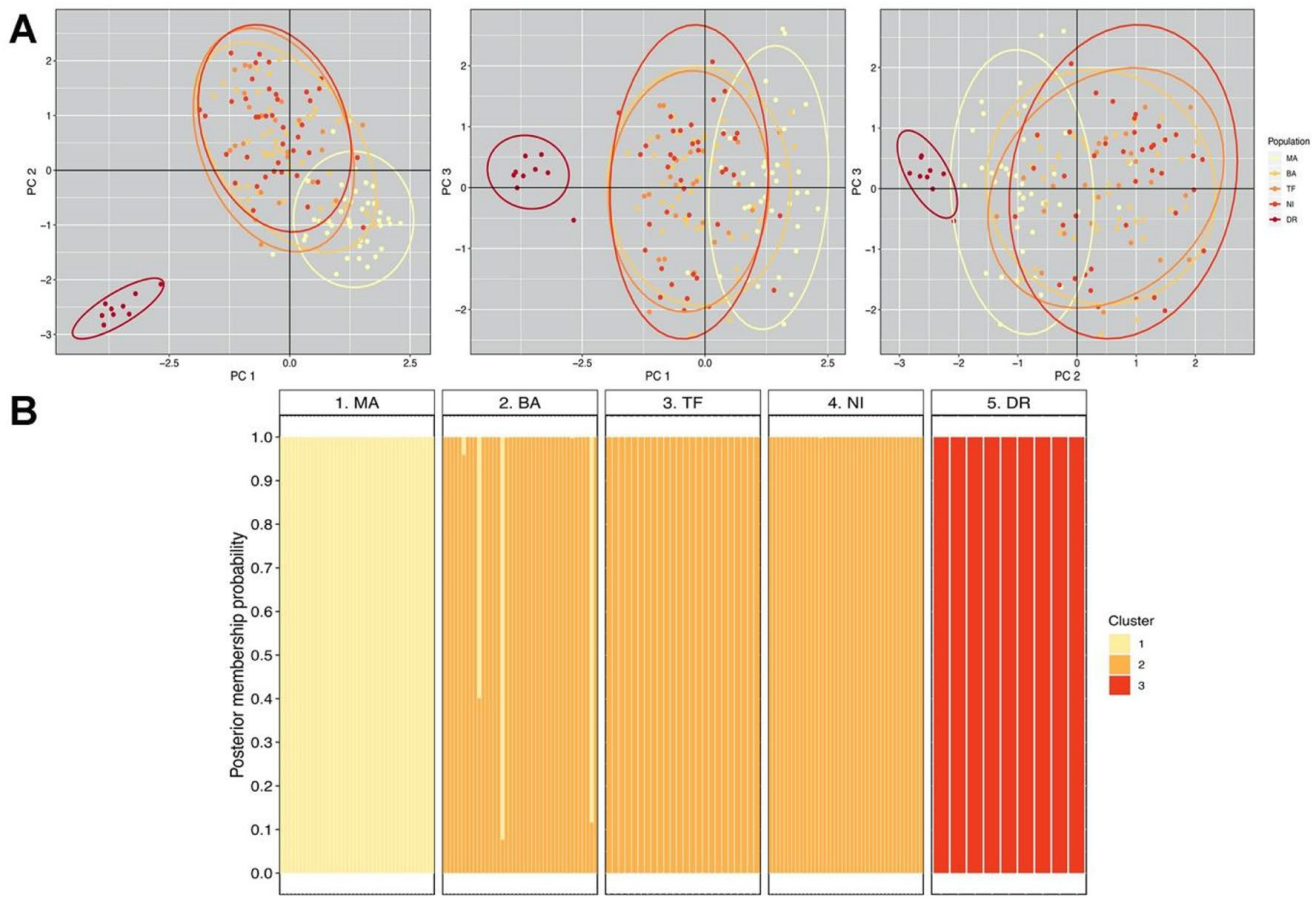
Genetic variation among five populations of *Aphrastura*, based on 153 individuals genotyped at 12 polymorphic microsatellite loci. (**A**) Scatterplot from the first three principal components that explained 25.5% of the genetic variance. 95% CI ellipses are shown. (**B**) Individual assignment to genetic clusters for the five sampled populations. Bars represent individual posterior membership probabilities to each of the three genetic clusters found using the ‘snapclust’ method. *MA* Manquehue, *BA* Bariloche, *TF* Tierra del Fuego, *NI* Navarino Island, *DR* Diego Ramírez (representing the proposed new species *A. subantarctica*) .

For the PCA analysis of the five sampled populations, we retained the first 43 dimensions, which explained 85% of the total genetic variation. A plot with the first three components (~ 26% of variation explained) showed that Navarino Island and the southern continental populations (Bariloche and Tierra del Fuego) formed a homogeneous group (Fig. 6). The northernmost continental population (Manquehue, central Chile) was slightly separated, but relatively well mixed with the southern continental group (Fig. 6). In contrast, the Diego Ramírez population appeared well isolated from all other sampled localities, regardless of the combination of principal components being examined (Fig. 6).

According to the AIC values obtained from the ‘snapclust’ method, the optimal number of genetic clusters within the group of sampled individuals was three (Supplementary Information Appendix 3). This estimate is congruent with the number of clusters emerging from the PCA analysis: (1) a central cluster, comprising the Manquehue population in the center of the species’ distributional range; (2) a southern cluster, composed of Bariloche, Tierra del Fuego Island, and Navarino Island populations; and (3) Diego Ramírez. The pairwise G-statistics indicated that differentiation between the central and southern clusters was low to moderate (Table 5), whereas Diego Ramírez showed strong differentiation from the latter two, regardless of the *G*_*ST*_ index being considered (Table 5). The posterior membership probabilities calculated from the ‘snapclust’ showed that some individuals were assigned to a population different from the one from which they were captured. This was true for some birds in the central and southern clusters, but not for any individual from Diego Ramírez (100% correct assignment; Fig. 6). These results are in line with the analysis using GeneClass2, in which no first-generation migrants were detected in Diego Ramírez for any of the 10 replicates performed, whereas the southern cluster harbored four potential immigrants from the central cluster (Supplementary Information Appendix 4).

**Table 5:** Pairwise G-statistics for the three main genetic clusters of *Aphrastura* populations identified (see “Methods” for details).

Genetic cluster			
	Central	South	Diego Ramírez
Central	–	*0.216*	*0.688*
South	0.055	–	*0.590*
Diego Ramírez	0.346	0.289	–

Values below the diagonal correspond to the Nei’s standardized index *G′*_*ST(Nei)*_, while values above the diagonal (italics) were calculated using Hedrick’s standardized index corrected for small samples *G″*_*ST*._ The true *G*_*ST*_ value is supposed to lie in between the two indexes^71^. All p values < 0.001 (based on 1000 permutations).

### Characterization of the new species

Based on the strong genetic differentiation, as well as marked morphological and behavioral differences described above for the Diego Ramírez *Aphrastura* population, as compared to the continental *Aphrastura* population, we propose the following new bird species.

Order: Passeriformes (Linnaeus, 1758)^47^.

Family: Furnariidae (Gray, 1840)^48^.

Genus: *Aphrastura* (Oberholser, 1899)^49^.

Scientific name: *Aphrastura subantarctica, *sp.* nov.* R Rozzi, CS Quilodrán, E Botero-Delgadillo, RD Crego, C Napolitano, O Barroso, JC Torres-Mura & RA Vásquez.

Common name (English): Subantarctic Rayadito.

Common name (Spanish): Rayadito Subantártico.

Diagnosis: Morphology.—Typical *Aphrastura* structure with rounded wings, and an idiosyncratic tail morphology. *Aphrastura*’s distal third of the inner web of the rectrices is abruptly and deeply excised, giving the tips of the feathers a thornlike appearance. No other genus in the family has a similar tail structure. *Aphrastura* differs in these morphological characters from the phylogenetically closest related genera in the subfamily Synallaxinae present in southwestern South America: *Leptasthenura* and *Sylviorthorhynchus*^50,51^. In contrast to *Aphrastura*, *Leptasthenura*’s tail is not abruptly and deeply excised at the distal portion of the inner web of the rectrices; in *Sylviorthorhynchus*, the rectrices are denuded of barbs^1^. *A. subantarctica* differs from *A. spinicauda*, in having on average a larger and heavier body (~ 25%), a larger beak (~ 15%), a larger tarsus (~ 5%), and a shorter tail (~ 16%) (Fig. 3; Supplementary Information Appendix 5 and 6). The primaries and secondaries are greyish on the ventral side with whitish edges; the central rectrices are dark grey on the ventral side, but do not differ between the two species. Color terms using Munsell Color’s^52^ notation are shown in Appendix 7 (Supplementary Information).

Holotype.—Adult male, Museo Nacional de Historia Natural, in Santiago, Chile (MNHNCL/AVE Nº 5439), collected on 22 July 2017 by OB and JCTM on Gonzalo Island, Diego Ramírez Archipelago, Magallanes and Antarctic region, Chile, at 56.52141°S 68.71647°W.

Description of holotype.—Color descriptions follow Vaurie^1^. Crown, hind neck and face sooty black, divided by a broad and conspicuous dull orange superciliary streak that extends to the back and merges with the brown color of the back. Other upperparts are fulvous brown, with dusky dark edges becoming rufous and uniform on the rump. Underparts whitish on the throat, breast, and abdomen becoming fulvous on the flanks and undertail coverts. No brood patch; no cloacal protuberance, little fat (1), no evidence of molt on head, body, wing, or tail.

Measurements of holotype.—Total length 145 mm; head and bill length 32.4 mm; exposed culmen 12.1 mm; bill depth 3.4 mm; bill width 3.4 mm; wing chord 59 mm; tarsus length 21.8 mm; tail length along central rectrices 66 mm; mass 15.1 g.

Paratypes (two).—(1) Juvenile female, MNHNCL/AVE 5440, collected 22 July 2017 at Gonzalo Island, Diego Ramírez Archipelago by OB and JCTM; same coloration as the adult, tarsus and gape yellowish; white tips in primaries and secondaries. No brood patch; no cloacal protuberance, no fat (0), no head, body, wing, or tail molt. Total length 150 mm; head and bill length 33.6 mm; exposed culmen 12.6 mm; bill depth 3.6 mm; bill width 3.2 mm; wing chord 63 mm; tarsus length 23 mm; tail length along central rectrices 69 mm; mass 15.9 g.—(2) Juvenile male, MNHNCL/AVE 5441, collected 22 July 2017 on Gonzalo Island, Diego Ramírez Archipelago by OB and JCTM; same coloration as the adult, gape and tarsus yellowish; white tips in primaries and secondaries. No brood patch; no cloacal protuberance, no fat (0), no head, body, wing, or tail molt. Total length 150 mm; head and bill length 33.6 mm; exposed culmen 12.9 mm; bill depth 3.5 mm; bill width 3.3 mm; wing chord 59 mm; tarsus length 23 mm; tail length along central rectrices 72 mm; mass 15.9 g.

Etymology. The specific epithet refers to the distribution restricted to the remote sub-Antarctic Magellanic ecoregion of Chile. By naming the new species after this region, one of the last pristine regions in the world^53^, the authors encourage the adoption of this small bird as a symbol for the conservation of this unique environment.

## Discussion

Oceans constitute a strong dispersal barrier for terrestrial animals but also for birds^54^. For small birds, such as *Aphrastura* spp*.*, an island separated from the closest island (Horn Island) by 111 km in one of the world’s windiest oceans^12^, can limit exchange and hence favor population differentiation. One of the best-studied examples on islands separated by short distances is the radiation of the Darwin’s finches (*Geospiza* spp.) on the Galapagos Islands in the Pacific Ocean^55^. We studied several populations of *Aphrastura spinicauda*, a year-round resident of forested habitats that rarely engages in long-distance flights^56–60^. While several populations are located on southern islands, our results suggest they are not reproductively isolated, except for the population on Diego Ramírez. This population is genetically differentiated from populations inhabiting the closest islands on Tierra del Fuego and the mainland, with no evidence of recent gene flow. This may explain the morphological divergence of the population in terms of body weight, beak shape, and tarsus and tail length. In addition, the distinct environmental conditions on Diego Ramírez, in particular the lack of trees or any other woody plants, strong winds, and the presence of dense tussock used by burrow-nesting seabirds, might have triggered behavioral divergence related to living in a herbaceous environment, such as nesting in cavities below or close to ground level. This markedly contrasts with the tree creeping behavior and cavity nesting displayed by their continental relatives inhabiting forest ecosystems.

### Morphological and behavioral differentiation

Individuals of *Aphrastura* on Diego Ramírez are on average heavier than their continental counterparts, and their tails are shorter. These morphological shifts might represent an adaptation to shorter and lower flights in dense tall grass habitats and windy conditions. These birds tend to fly mostly short distances among the *Poa flabellata* tussocks, which protect them from the strong winds. These movement patterns, shaped by the habitat conditions, conspicuously contrast with those of individuals from continental populations that move within the understory (< 1 m) to the top of the canopy (> 15 m), and often fly into open spaces while crossing gaps between tree patches^7^. On Diego Ramírez, restricted movement within a more sheltered habitat, close to the ground level, may have driven selection for shorter tails that may facilitate moving among dense tussock. Shorter tails could also have arisen through genetic drift after a small number of birds reached the archipelago or because other selective pressures for longer tails might have been relaxed in the environmental conditions of Diego Ramírez. A larger body size is typically found in birds that have colonized islands, and is a well-documented pattern of convergent evolution^61^. This has been described as the “island syndrome”, in which small birds (< 1 kg) are predicted to increase their body size in response to reduced predation pressure and food limitation under the new insular conditions^62^. Selection on fast escape from predators may be relaxed on the archipelago, as terrestrial predators are absent^63^. In addition, a larger body size may have been favored on Diego Ramírez because it increases thermoregulation efficiency^64^. While a larger body size has also been described in the subspecies (*Aphrastura s. bullocki*)^13^ and species (*Aphrastura masafuerae*)^2,65,66^ of *Aphrastura* inhabiting islands, the behavior related to grass habitat use and nesting at ground level seems to be unique to *Aphrastura* on Diego Ramírez. Another subspecies inhabiting Patagonian islands is *Aphrastura s. fulva*, but because the main differences from continental *Aphrastura spinicauda* are related to color patterns^13^, it is also expected to be morphologically and behaviorally different from *Aphrastura* on Diego Ramírez. The findings of our study are thus consistent with the suggestion made by François Vuilleumier thirty years ago^67^, who characterized *Aphrastura spinicauda* as a geographically variable species. He also pointed out that the individuals observed on Diego Ramírez were morphologically and behaviorally different from northern populations, and more similar to wrens (Troglodytidae) than to species ecologically equivalent to *A. spinicauda*, such as titmice (Paridae) or treecreepers (Certhiidae).

The population on Diego Ramírez uses a distinct habitat that lacks woody plants, in ways that are unusual when compared to the continental populations. The lack of trees with nesting cavities has led to an association with breeding seabirds, which nest on Diego Ramírez during the austral spring and summer. *Aphrastura* individuals use burrows of different seabird species, sometimes associating with larger seabirds (e.g., Grey-headed albatross), which may provide protection from weather and potential aerial predators (e.g., Striated caracara *Phalcoboenus australis* and the Chilean skua *Stercorarius chilensis*). Across the entire range of *Aphrastura spinicauda*, individuals respond to intruders near their nest sites with alarm calls. In the continental populations, mobbing calls have minimum and maximum frequencies of 2.82 and 13.01 kHz, respectively, with at least six notes per second in central and southern Chile^68^. However, based on a previous record in 2001, it seems that minimum and maximum frequencies of mobbing calls of *Aphrastura subantarctica* are lower, with the same number of notes (see^69^). While these preliminary observations have to be confirmed by future studies, the low call frequencies of *Aphrastura* on Diego Ramírez could be related to the high ambient noise, as well as their larger body size^70^.

### Genetic differentiation

The genetic structure resulting from both mitochondrial and autosomal data suggests that the *Aphrastura* population from Diego Ramírez represents an isolated entity. The autosomal differentiation is substantial even when considering values of the *G′*_*ST(Nei)*_ index, which is a more conservative statistic than Hedrick’s index^71^. Despite sharing a mitochondrial haplotype with populations from Navarino and the Horn islands, the *Aphrastura* population of Diego Ramírez is also differentiated at the mitochondrial level, as shown by the significant genetic distance between them and individuals in Navarino and the Horn islands. Furthermore, Diego Ramírez appeared as a genetically separated cluster in our analyses, indicating a complete absence of subsequent, or at least recent, migration to or from the archipelago. When excluding the Diego Ramírez individuals, the other populations of *A*. *spinicauda* have no clear genetic structure at the autosomal and mitochondrial level across their distribution, which is consistent with moderate to high levels of gene flow, as documented by other authors^30^. Our results support the recent observation of the absence of current gene flow between the Diego Ramírez population and their relatives inhabiting the continent and islands closer to it^10^. This population could thus be considered a separate evolutionary unit.

The sub-Antarctic Magellanic ecoregion experienced variations in sea level and volume of ice sheet during the Last Glacial Maximum (LGM, 18,000–28,000 years BP)^72,73^, which has resulted in a heterogeneous environment with high level of endemism across various biological groups^74^. The population on Diego Ramírez was probably established from a unique colonization event of a small group of individuals that originated from the southern Patagonian islands, which are geographically nearest to Diego Ramírez, such as Navarino or Horn islands, with one shared mitochondrial haplotype among them suggesting this historical event. In addition, the presence of a single *cytb* haplotype on Diego Ramírez suggests a strong founder effect, while the absence of new private haplotypes suggests a scenario of recent, post-LGM colonization of Diego Ramírez. The noticeably low levels of heterozygosity, allelic richness, and number of private alleles in the microsatellite data further supports this hypothesis. In evolutionary terms, the relatively short time that has passed since a post-LGM colonization event might have been sufficient for the Diego Ramírez population to genetically differentiate from *A*. *spinicauda*. The absence of the main mitochondrial haplotype present in all *A*. *spinicauda* populations, from the northernmost to the southernmost ones, suggests that the population on Diego Ramírez is undergoing a peripatric divergence from *A*. *spinicauda.*

### A new taxonomic unit

Our analyses indicate that the *Aphrastura* population from Diego Ramírez compose a demographically independent population that should be considered as a new conservation and taxonomic unit. This population has remained isolated and encompasses specific morphological, genetic, and behavioral features, for which reason it is now proposed as a new species^75^. In his influential paper, de Queiroz^76^ reconciled the concept of species and stated that the only nonnegotiable condition for a species to be considered as such is to separately evolve from another lineage, which according to our data has taken place for the *Aphrastura* population in Diego Ramírez. Whether there is complete lineage sorting and intrinsic reproductive isolation among the *Aphrastura* populations or not will only show how far the diverging process has gone through the speciation continuum (e.g.,^77^).

We have presented multiple pieces of evidence suggesting the existence of a new species by combining morphological, ecological and genetic data, and we argue that this integrative framework is crucial for defining new taxonomic units. Acoustic analysis, a powerful tool for supporting species limits in suboscine passerines, including Furnariidae^51^, may provide more evidence of differentiation of the Diego Ramírez population. Further research may include samples from *A. masafuerae* and *A. s. bullocki*, as well as full genomes for evaluating the genomic landscape of divergence during the speciation process of the genus *Aphrastura*. Several other bird taxa with small genetic differences have been reclassified and subsequently considered as valid species, such as the common swift (*Apus apus*) and pallid swift (*A. pallidus*)^78^, as well as the greater spotted eagle (*Clanga clanga*) and lesser spotted eagle (*C. pomarine*)^79^, among others (see^80^).

## Concluding remarks

We propose *A. subantartica* as a new species. The genetic, morphological, and ecological divergence of this population, which may have resulted from isolation on an island with a distinct habitat, is probably an ongoing evolutionary process. Because of the small size of the Diego Ramírez islands and the potential arrival of exotic mammal predators, it is pressing to protect this new endemic species from extinction. The Diego Ramírez Archipelago encompasses the southernmost extreme islands of the American continent and is free of invasive alien species. Measures should be put into place to keep exotic mammals, such as rats (*Rattus rattus*), domestic cats (*Felis catus*), and American minks (*Neovison vison*)–which are all present on other islands of the Cape Horn Biosphere Reserve^24,81^–off the Diego Ramírez islands. In particular, the rapid expansion of the American mink has impacted bird populations on other subantarctic islands that have evolved in the absence of terrestrial mammal predators^82,83^. Collaboration between the scientific community and other institutions, in particular the Chilean Navy, which has been regularly present on this archipelago since the establishment of the lighthouse on Gonzalo island in 1951, is critical for the success of scientific long-term monitoring and conservation programs^9^. In 2019, the Diego Ramírez-Drake Passage Marine Park was created by the Chilean government to protect one of the few archipelagos that is still free from the arrival of exotic species worldwide^12^. The description of *A. subantarctica* is also an appeal to the community to protect not only a population of a new species, but also to protect a remote natural laboratory that represents an opportunity to preserve the diversity of nature and its ecological and evolutionary processes.

## Supplementary Information

Supplementary Information.

## Data Availability

The dataset used in the analysis is publicly available on zenodo: 10.5281/zenodo.6983420.
