# Supplementary material for: The Subantarctic Rayadito (Aphrastura subantarctica), a new bird species on the southernmost islands of the Americas
Source: Sci Rep. 2022 Aug 26;12:13957. doi: 10.1038/s41598-022-17985-4 (PMC9418250; doi:10.1038/s41598-022-17985-4)
Supplement: Supplementary file 1 — Supplementary Information. [file 41598_2022_17985_MOESM1_ESM.pdf]

Supplementary Information

The Subantarctic Rayadito (*Aphrastura subantarctica*), a new bird species on the southernmost islands of the Americas

Ricardo Rozzi[1,2*], Claudio S. Quilodrán[1,3*], Esteban Botero-Delgadillo[4,5], Constanza Napolitano[1,6,7], Juan Carlos Torres-Mura[1,8], Omar Barroso[1], Ramiro Daniel Crego[9], Camila Bravo[5], Silvina Ippi[10], Verónica Quirici[11], Roy Mackenzie[1], Cristián G. Suazo[1,12], Juan Rivero-de-Aguilar[1], Bernard Goffinet[1,13], Bart Kempenaers[4], Elie Poulin[1,5,14] & Rodrigo A. Vásquez[1,5]

[1]Cape Horn International Center (CHIC), Parque Etnobotánico Omora, Universidad de Magallanes, Puerto Williams, Chile

[2]Sub-Antarctic Biocultural Conservation Program, Department of Philosophy and Religion & Department of Biological Sciences, University of North Texas, Denton, TX, USA

[3]Department of Biology, University of Fribourg, Fribourg, Switzerland.

[4]Department of Behavioural Ecology and Evolutionary Genetics, Max Plank Institute for Ornithology, Seewiesen, Germany.

[5]Departamento de Ciencias Ecologicas, Facultad de Ciencias, Universidad de Chile, Santiago, Chile

[6]Departamento de Ciencias Biológicas y Biodiversidad, Universidad de Los Lagos, Osorno, Chile

[7]Instituto de Ecología y Biodiversidad, Santiago, Chile

[8]AvesChile (Unión de Ornitólogos de Chile), Santiago, Chile

[9]Smithsonian National Zoo and Conservation Biology Institute, Conservation Ecology Center, 1500 Remount Rd, Front Royal, VA 22630, USA

[10]Departamento de Zoología, CRUB Universidad Nacional del Comahue – CONICET, Bariloche, Argentina

[11]Centro de Investigación para la Sustentabilidad, Facultad de Ciencias de la Vida, Universidad Andres Bello, Santiago, Chile

[12]Department of Animal Ecology and Systematics, Justus Liebig University Giessen, Giessen, Germany

[13]Department of Ecology and Evolutionary Biology, University of Connecticut, Storrs, CT 06269 USA

[14]Millennium Institute Biodiversity of Antarctic and Subantarctic Ecosystems (BASE), Facultad de Ciencias, Universidad de Chile, Santiago, Chile

[*]Corresponding authors: ricardo.rozzi@unt.edu; claudio.quilodran@unifr.ch

Supplementary Information

Appendix 1: Morphological diagnosis of *Aphrastura* species and subspecies

Aphrastura s. fulva is endemic to Chiloé Island and Chonos islands, which have extensive areas of Valdivian and North-Patagonian forests, dominated by species of *Nothofagus* and *Aextoxicon punctatum*[1]. Johnson and Goodall [2] distinguished *A. s. fulva* from the continental subspecies by its cinnamon (*fulvus*, in Latin) plumage on the throat, chest and belly. However, Moreno, et al. [3] found that in Chiloe there is continuous variation from the whitish to the reddish-yellow throat, and suggested that further studies are needed to confirm the systematic status of this subspecies.

Aphrastura s. bullocki is endemic to Mocha Island (34.3 km off the mainland), an area of 48 km^2 covered mostly by native forests dominated by *A. punctatum*, *Drimys winteri*, and several species of Myrtaceae[4]. This subspecies distinguishes by having white plumage on the throat and upper part of the chest, and ochraceous buff in the lower parts of the chest and belly, particularly towards the flanks[5,6]. *A. s. bullocki* has also a larger size, longer wings, and greater weight[2].

Aphrastura masafuerae was first described based on its marked morphological differentiation from mainland rayaditos. As compared to *A. spinicauda*, *A. masafuerae* has a considerably bigger and coarser body size and longer tarsus[7-9], a pattern found in many island species (see[10,11]). The beak is also longer and more robust, with a distinctively yellow lower mandible[12]. Although its wings and tail retain a similar coloration to *A. spinicauda*, the plumage of its head, throat, chest, belly and upper back has a characteristic grayish color. The morphological differentiation of this species was later supported by molecular analysis[13]. *A. masafuerae* inhabits over 600 m altitude on Alejandro Selkirk Island (formerly Masafuera), in forest areas dominated by broadleaf evergreen trees of *Drimys confertifolia*, and the arboreal fern *Dicksonia externa*[14].

Supplementary Information

Appendix 2: Nesting sites of the *Aphrastura* population from Diego Ramírez. Breeding couples nested in different altitudinal ranges of the islands, including sites closer to the coast, characterized by mixed coverage of tusssok and rocky substratum (left figure panel), as well as sites in higher and wind-exposed areas of the islands, 100% covered by the tussock. The breeding activity also displayed associations with seabirds, such as a cavity nest observed in the base of an active nest of Grey-headed albatross in the summer 2020-2021 (right figure panel). Images by Maximiliano Daigre.

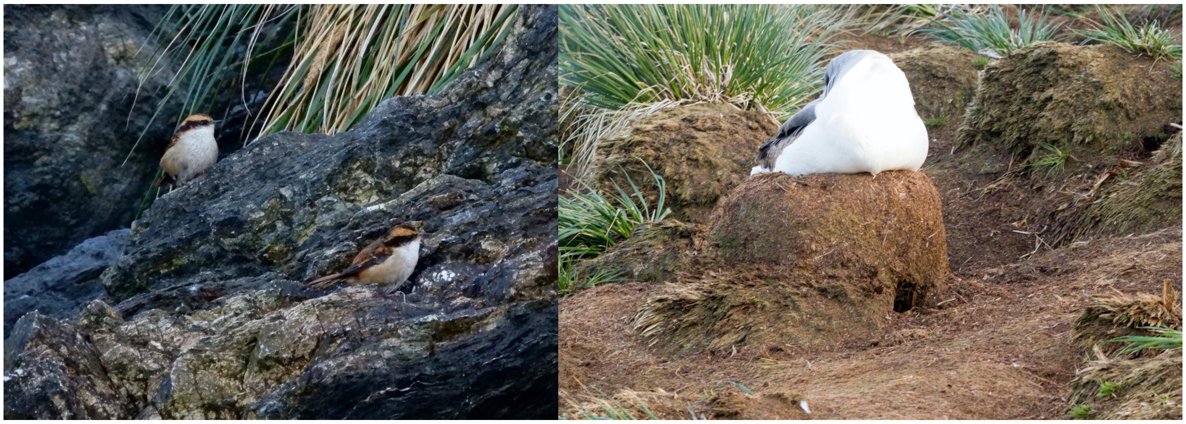

Supplementary Information

Appendix 3: Results from the 'snapclust' analysis used to estimate the number of genetic clusters (k) among five sampled populations of Thorn-tailed Rayadito (*Aphrastura spinicauda*). The optimal number of clusters was determined using the Akaike Information Criterion (AIC).

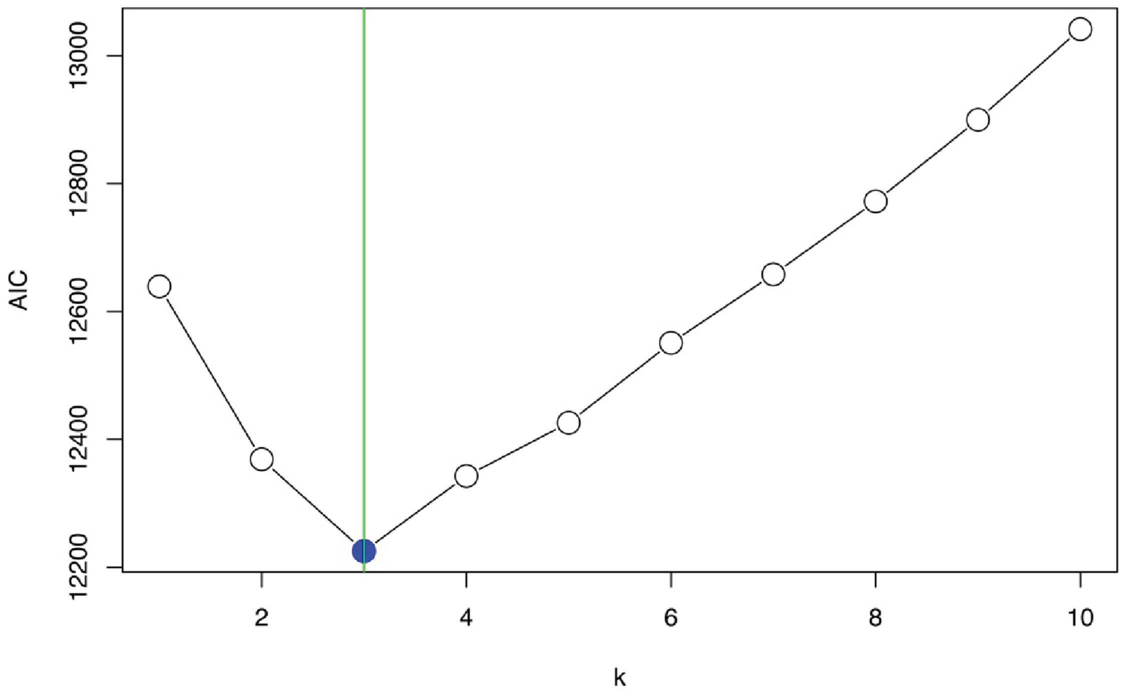

Supplementary Information

Appendix 4: Results from analysis using GeneClass2 to identify first-generation migrants. Detection of migrants was carried out using Paetkau's et al.[15] criterion for likelihood computation (L_h) and Paetkau's et al.[16] resampling method for probability calculation, setting the default frequency for missing alleles (0.01) and 10 replicates with 100 simulated individuals each ($\alpha = 0.01$). Given that results from all 10 replicates suggested the same four individuals as potential migrants, only results from the first simulation are presented here. The threshold p value for the analysis was set to 0.01. The lowest -log(L) value indicates the most likely population of origin for any given individual. Rows in bold letters represent potential first-generation migrants.

Original population	Home -log(L)	Home probability	Center '-log(L)	South '-log(L)	Diego Ramírez '-log(L)	Loci
Center	0	0.5	14.024	19.284	27.964	12
Center	0	0.5	16.308	21.588	28.712	12
Center	0	0.5	13.94	21.351	31.453	12
Center	0	0.51	13.459	15.693	26.105	12
Center	0	0.5	13.494	19.976	30.291	12
Center	0	0.5	16.37	27.59	34.659	12
Center	0	0.5	15.537	19.569	34.897	12
Center	0	0.5	20.479	27.468	37.617	12
Center	0	0.5	17.285	21.09	33.499	12
Center	0	0.5	18.9	20.498	32.349	12
Center	0	0.5	17.922	24.783	29.785	12
Center	0	0.5	18.501	21.043	32.78	12
Center	0	0.5	17.88	23.678	35.057	12
Center	0	0.5	13.617	18.68	31.7	12
Center	0	0.5	15.309	19.575	29.189	12
Center	0	0.5	18.783	24.113	32.675	12
Center	0	0.5	16.946	20.497	34.571	12
Center	0	0.5	15.295	16.618	23.918	12
Center	0	0.5	16.06	18.661	29.895	12
Center	0	0.5	15.115	21.277	31.127	12
Center	0	0.5	16.196	23.015	33.479	12
Center	0	0.5	20.4	23.728	38.927	12
Center	0	0.5	16.878	19.346	24.59	12
Center	0	0.5	15.353	20.961	33.846	12

Supplementary Information

Center	0	0.5	15.95	20.823	30.596	12
Center	0	0.5	18.496	20.074	36.871	12
Center	0	0.5	14.091	18.661	33.289	12
Center	0	0.5	15.563	19.742	32.571	12
Center	0	0.5	14.157	21.121	36.127	12
Center	0	0.5	13.872	18.518	28.818	12
Center	0	0.5	14.15	17.948	30.503	12
Center	0	0.5	13.623	18.904	29.604	12
Center	0	0.5	17.756	18.853	29.754	12
Center	0	0.51	15.51	22.834	29.992	12
Center	0	0.5	18.652	29.138	38.148	12
Center	0	0.5	13.731	18.818	29.337	12
Center	0	0.5	15.489	20.37	27.926	12
Center	0	0.5	17.55	21.526	34.525	12
Center	0	0.5	15.505	19.591	32.407	12
Center	0	0.5	13.329	20.929	31.013	12
South	0	0.51	22.743	18.027	34.571	12
South	0	0.52	23.308	22.019	29.067	12
South	0	0.52	20.985	16.544	27.756	12
South	0	0.52	21.258	16.627	28.117	12
South	0	0.52	20.169	20.026	28.687	12
South	0	0.52	20.263	17.153	29.233	12
South	0	0.51	24.99	16.794	25.73	12
South	0	0.51	20.143	15.994	23.594	12
South	0	0.51	17.784	15.96	34.233	12
South	0	0.52	25.177	23.657	36.205	12
**South**	**2.286**	**0**	**16.758**	**19.044**	**31.78**	**12**
South	0	0.52	16.907	15.649	26.633	12
South	0	0.51	23.06	18.595	29.12	12
South	0	0.52	21.822	16.953	30.956	12
South	0	0.51	23.233	19.83	26.596	12
South	0	0.52	25.042	17.601	25.668	12
South	0	0.52	19.386	18.312	30.633	12
South	0	0.52	23.474	19.976	29.517	12
South	0	0.52	23.913	16.563	32.081	12
**South**	**2.416**	**0**	**20.478**	**22.894**	**33.55**	**12**
South	0	0.52	20.811	18.5	28.515	12
South	0	0.52	24.172	20.96	25.027	12
South	0	0.52	21.747	16.694	28.21	12
South	0	0.52	22.189	19.511	29.559	12
South	0	0.51	22.41	17.003	30.399	12
South	0	0.51	23.054	22.59	31.607	12
South	0	0.51	25.005	20.639	28.053	12

South	0	0.52	28.29	21.932	32.436	12
South	0	0.52	21.122	17.219	29.691	12
South	0	0.52	19.627	18.725	27.95	12
South	0	0.52	17.535	17.085	33.358	12
South	0	0.52	25.196	16.53	24.758	12
South	0	0.52	21.338	19.998	31.214	12
**South**	**1.365**	**0.01**	**22.395**	**23.76**	**32.853**	**12**
South	0	0.51	17.88	16.356	27.092	12
South	0	0.52	20.038	16.98	28.005	12
South	0	0.52	22.659	16.534	28.922	12
South	0	0.52	21.774	19.84	33.517	12
**South**	**1.129**	**0.01**	**16.236**	**17.365**	**31.7**	**12**
South	0	0.51	20.532	15.61	22.402	12
South	0	0.52	23.252	18.549	33.081	12
South	0	0.52	21.504	17.401	33.275	12
South	0	0.52	17.873	16.365	28.356	12
South	0	0.52	21.317	14.773	19.712	12
South	0	0.51	24.59	22.804	26.718	12
South	0	0.51	20.678	17.163	24.364	12
South	0	0.51	15.761	14.73	27.565	12
South	0	0.52	18.537	17.64	30.737	12
South	0	0.52	20.45	15.221	28.742	12
South	0	0.52	22.244	18.562	38.872	12
South	0	0.52	17.571	14.747	18.996	12
South	0	0.52	20.897	18.713	24.251	12
South	0	0.52	22.199	14.242	25.582	12
South	0	0.52	21.04	16.722	27.705	12
South	0	0.51	20.205	15.964	24.381	12
South	0	0.51	19.958	15.575	25.993	12
South	0	0.52	20.678	18.686	27.964	12
South	0	0.52	18.259	16.482	27.47	12
South	0	0.52	17.333	17.059	31.268	12
South	0	0.52	17.595	15.495	24.964	12
South	0	0.52	27.703	20.606	32.144	12
South	0	0.51	24.369	17.923	29.558	12
South	0	0.52	20.245	13.403	26.022	12
South	0	0.52	21.189	15.152	23.894	12
South	0	0.51	21.56	17.748	27.561	12
South	0	0.52	18.871	12.716	28.285	12
South	0	0.52	24.21	16.516	29.291	12
South	0.439	0.01	16.721	17.16	29.754	12
South	0	0.52	24.072	20.956	36.994	12
South	0	0.52	19.796	17.07	28.808	12

South	0	0.52	17.218	13.683	26.432	12
South	0	0.51	20.995	16.134	30.081	12
South	0	0.52	22.927	14.847	29.221	12
South	0	0.52	20.873	16.947	29.859	12
South	0	0.52	18.835	16.245	34.494	12
South	0	0.52	22.471	17.047	23.42	12
South	0	0.52	17.767	13.697	22.579	12
South	0	0.52	22.091	15.109	26.49	12
South	0	0.52	21.399	16.45	23.787	12
South	0	0.52	19.418	19.116	28.816	12
South	0	0.52	21.758	18.834	30.811	12
South	0	0.52	25.614	14.274	21.265	12
South	0	0.52	18.745	15.604	27.148	12
South	0.39	0.02	18.274	18.664	30.638	12
South	0	0.52	18.585	16.338	22.888	12
South	0	0.52	19.345	15.846	27.26	12
South	0	0.51	24.501	19.319	26.918	12
South	0	0.52	22.52	17.515	30.022	12
South	0	0.52	20.201	14.896	28.939	12
South	0	0.52	20.229	16.061	26.088	12
South	0	0.52	24.116	18.508	28.57	12
South	0	0.52	27.187	18.218	24.846	12
South	0	0.52	16.953	14.163	20.725	12
South	0	0.52	19.439	14.705	27.691	12
South	0	0.51	18.458	15.148	30.592	12
South	0	0.52	20.093	13.181	27.059	12
South	0	0.52	22.786	15.896	32.621	12
South	0	0.52	21.258	16.746	28.726	12
South	0	0.52	20.257	14.742	33.111	12
South	0	0.52	18.84	16.224	27.42	12
South	0	0.52	22.867	16.267	28.604	12
South	0	0.51	21.469	18.047	31.348	12
South	0	0.52	18.558	15.773	36.013	12
South	0	0.52	19.889	15.898	28.922	12
Diego Ramírez	0	0.5	26.574	18.127	4.194	12
Diego Ramírez	0	0.5	25.028	16.95	3.171	12
Diego Ramírez	0	0.5	22.971	17.054	6.419	12
Diego Ramírez	0	0.5	23.892	17.479	4.118	12
Diego Ramírez	0	0.5	24.678	16.555	3.268	12
Diego Ramírez	0	0.5	24.213	16.991	4.921	12
Diego Ramírez	0	0.5	24.449	19.369	9.818	12
Diego Ramírez	0	0.5	23.903	17.975	5.076	12
Diego Ramírez	0	0.5	25.301	16.905	3.192	12

Supplementary Information

Appendix 5: Phenotypic comparisons among populations of Aphrastura *spinicauda* at Omora Park on Navarino Island and the North West Arm of the Beagle Channel, and of *A. subantarctica* from Gonzalo Island (Diego Ramírez Archipelago).

	**Omora (Navarino)**			**Pia (Beagle NW)**			**Diego Ramirez**		
**Measurements**	**Mean ± SD**	**Min-Max**	**N**	**Mean ± SD**	**Min-Max**	**N**	**Mean ± SD**	**Min-Max**	**N**
Tail length (mm)	70.6 ± 4.7	55 - 79	54	69.7 ± 6.0	47 - 79	50	58.6 ± 11.0	41 – 72	13
Tarsus length (mm)	20.6 ± 0.6	19.2 - 21.9	54	21.3 ± 1.2	19.0 - 24.5	50	22.0 ± 0.7	20.4 - 22.9	13
Wing length (mm)	60.1 ± 2.6	55 - 65	54	59.1 ± 2.4	55 - 66	50	60.3 ± 1.9	56 - 63	13
Beak length(mm)	10.0 ± 0.9	7.4 - 11.6	54	9.9 ± 1.0	8.2 - 13.6	50	11.7 ± 0.7	10.1 - 12.8	13
Beak width (mm)	2.9 ± 0.3	2.3 - 3.7	54	2.7 ± 0.4	1.9 - 3.4	50	3.4 ± 0.2	3.1 - 3.8	13
Weight (g)	12.5 ± 1.1	10.0 - 14.9	54	12.1 ± 1.2	10.2 – 15.0	50	16.3 ± 1.5	14.9 - 19.3	13
Nest location	Tree cavities			Tree cavities			Ground cavities, below tussock grass		

Supplementary Information

Appendix 6: Color painting illustrations for *Aphrastura* taxonomic groups. The new species (A) from the Diego Ramírez archipelago (*A. subantarctica*) differs morphologically from (B) *A. spinicauda* by its larger beak, longer tarsus, shorter tail, and higher weight. Illustrations by Mauricio Alvarez Abel.

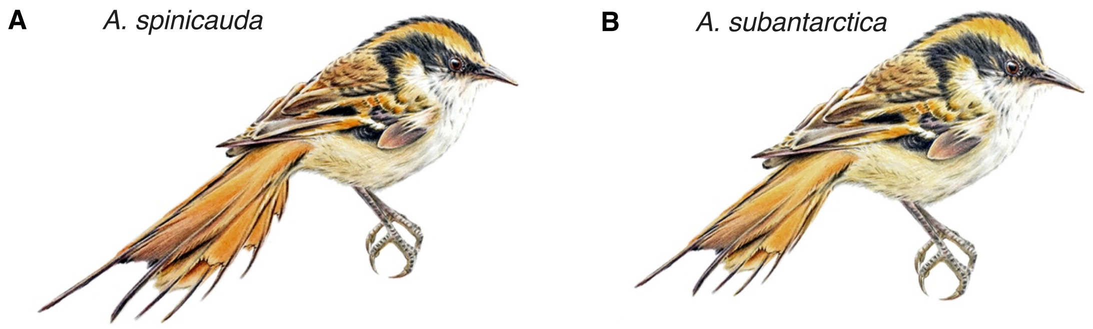

Supplementary Information

Appendix 7: Color characteristics of the feathers in different body parts. The holotype (MNHNCL/AVE 5439; adult male) and two paratypes – (MNHNCL/AVE 5440; juvenile female) and (MNHNCL/AVE 5441; juvenile male) – of *Aphrastura subantarctica* are presented. The notation follows Munsell[17].

**Body parts**	**MNHNCL/AVE 5439**	**MNHNCL/AVE 5440**	**MNHNCL/AVE 5441**
Crown	10YR 6/8	10YR 6/8	10YR 6/8
Occiput	10YR 6/8	10YR 6/8	10YR 6/8
Eyebrow	7.5 YR 6/8	7.5 YR 6/8	7.5 YR 6/8
Lesser coverts	7.5 YR 7/8	7.5 YR 7/8	7.5 YR 7/8
Breast	2.5 YR 8/1	2.5 YR 8/1	2.5 YR 8/1
Abdomen	10YR 8/1	10YR 8/1	10YR 8/1
Rump	10YR 3/3	10YR 3/3	10YR 3/3
Coverts	2.5 Y 3/1	2.5 Y 3/1	2.5 Y 3/1
Primaries	10YR 4/1	10YR 4/1	10YR 4/1
Primaries bands	10YR 4/4	10YR 4/4	10YR 4/4
Scapular spots	White 9.5/N	White 9.5/N	White 9.5/N
Internal edges of primaries	2.5 Y 8/2	2.5 Y 8/2	2.5 Y 8/2
Throat	White 9.5/N	White 9.5/N	White 9.5/N
Uppertail	7.5YR 4/3	7.5YR 4/3	7.5YR 4/3
Middle of tail	10YR 2/2	10YR 2/2	10YR 2/2
Tip of tail feathers	10YR 2/1	10YR 2/1	10YR 2/1
Tail ventral	7.5YR 6/2	7.5YR 6/2	7.5YR 6/2

Supplementary Information

Literature cited

1 Rozzi, R., Martínez, D., Willson, M. F. & Sabag, C. in *Ecología de los bosques nativos de Chile* (eds J. J. Armesto, C. Villagrán, & M. T. Kalin) 135-152 (Editorial Universitaria, 1996).

2 Johnson, A. W. & Goodall, J. *The birds of Chile and adjacent regions of Argentina, Bolivia and Peru*. (Platt Establecimientos Graficos, 1967).

3 Moreno, J., Merino, S., Vásquez, R. A. & Armesto, J. J. Breeding biology of the Thorn-tailed Rayadito (Furnariidae) in south-temperate rainforests of Chile. *Condor* **107**, 69–77 (2005).

4 Le-Quesne, C., Villagrán, C. & Villa, R. Historia de los bosques relictos de" olivillo"(Aextoxicon punctatum) y Mirtáceas de la Isla Mocha, Chile, durante el Holoceno tardío. *Revista Chilena de Historia Natural* **72**, 31-47 (1999).

5 Chapman, F. M. Descriptions of new birds from Mocha Island, Chile, and the Falkland Islands: with comments on their bird life and that of the Juan Fernandez Islands and Chiloe Island, Chile. American Museum novitates; no. 762. (1934).

6 Bullock, D. Las aves de la Isla de la Mocha. *Revista Chilena de Historia Natural* **39**, 232-253 (1935).

7 Philippi, R. & Landbeck, L. Beitrage zur Fauna Chiles. *Arch Naturgesch* **32**, 121-132 (1866).

8 Hahn, I. & Römer, U. New observations of the Masafuera Rayadito Aphrastura masafuerae. *Cotinga* **6**, 17-19 (1996).

9 Vaurie, C. Taxonomy and geographical distribution of the Furnariidae (Aves, Passeriformes). Bulletin of the AMNH; v. 166, article 1. (1980).

10 Clegg, S. M. & Owens, P. The 'island rule'in birds: medium body size and its ecological explanation. *Proceedings of the Royal Society of London. Series B: Biological Sciences* **269**, 1359-1365 (2002).

11 Lomolino, M. V. Body size evolution in insular vertebrates: generality of the island rule. *Journal of Biogeography* **32**, 1683-1699 (2005).

12 Hahn, I. & Römer, U. New observations of the Masafuera Rayadito Aphrastura masafuerae. *Cotinga* **6**, 17–19 (1996).

13 Gonzalez, J. Phylogenetic position of the most endangered Chilean bird: the Masafuera Rayadito (Aphrastura masafuerae; Furnariidae). *Tropical Conservation Science* **7**, 677-689 (2014).

14 Tomasevic, J. A., Hodum, P. J. & Estades, C. F. On the ecology and conservation of the critically endangered Masafuera Rayadito (Aphrastura masafuerae). *Ornitologia Neotropical* **21**, 535-543 (2010).

15 Paetkau, D., Calvert, W., Stirling, I. & Strobeck, C. Microsatellite analysis of population structure in Canadian polar bears. *Molecular ecology* **4**, 347-354 (1995).

16 Paetkau, D., Slade, R., Burden, M. & Estoup, A. Genetic assignment methods for the direct, real-time estimation of migration rate: a simulation-based exploration of accuracy and power. *Molecular ecology* **13**, 55-65 (2004).

17 Munsell Color Charts. *Munsell Soil Color Charts*. (Munsell Color Company, 2000).
